# Cost-effective, green HPLC determination of losartan, valsartan and their nitrosodiethylamine impurity: application to pharmaceutical dosage forms

**DOI:** 10.1098/rsos.220250

**Published:** 2022-06-01

**Authors:** Soad S. Abd El-Hay, Magda Elhenawee, Khaled Maged, Adel Ehab Ibrahim

**Affiliations:** ^1^ Analytical Chemistry Department, Faculty of Pharmacy, Zagazig University, Zagazig, Egypt; ^2^ Pharmaceutical Analytical Chemistry Department, Faculty of Pharmacy, Sinai University, El-Areesh, Egypt; ^3^ Pharmaceutical Analytical Chemistry Department, Faculty of Pharmacy, Port-Said University, Port-Said, Egypt; ^4^ Natural and Medical Sciences Research Center, University of Nizwa, P.O. Box 33, Birkat Al Mauz, Nizwa 616, Oman

**Keywords:** losartan, valsartan, nitrosodiethylamine, process-related impurities, green chromatography

## Abstract

Angiotensin-converting enzyme inhibitors are one of the most widely used anti-hypertensive drugs which are used to reduce hypertension. In 2018, the United States Food and Drug Administration together with the European Medicine Agency declared the presence of carcinogenic nitrosamine impurities such as nitrosodiethylamine (NDEA) in some of the products, including valsartan (VLS) and losartan (LOS), and drugs' recall procedures were started. Thus, they should be controlled to be below the acceptable cancer risk level to ensure safety of the pharmaceutical products. Therefore, sensitive and reliable analytical methods were required for detection and quantitation of NDEA in bulk and finished drug products. Green analytical chemistry has received great interest to minimize the amount of organic solvents consumed without loss in chromatographic performance. A green and sensitive HPLC method was developed for the determination of NDEA in LOS and VLS using mobile phase of 0.02 M ammonium acetate adjusted to pH 7.2 and ethanol in gradient manner. Limits of detection and limits of quantification for NDEA were estimated to be 0.2 and 0.5 µg ml^−1^, respectively. The standardized limits of NDEA impurity in drug substances were set as 0.56 ppm, which indicates the feasibility of its determination by the proposed conventional method without need for expensive instrumentations (e.g. MS/MS detectors) that are not found in most pharmaceutical quality control laboratories.

## 1. Introduction

**Figure 1.**
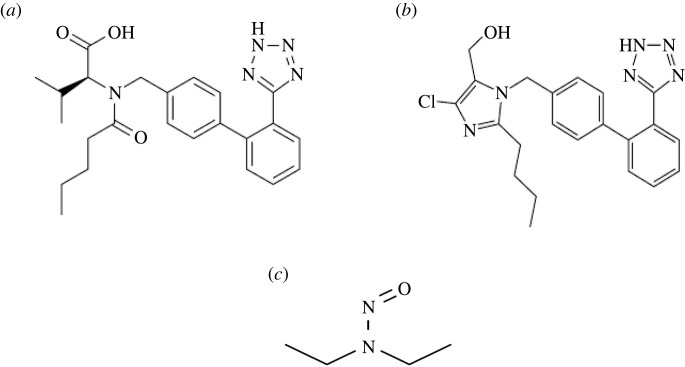
Chemical structures of (*a*) LOS, (*b*) VLS and (*c*) NDEA.

Hypertension is one of the most endemic diseases that threaten human beings in developed and developing countries. It is characterized by the increment of systolic and diastolic blood pressures, one of them or both above normal levels [1]. In 2015, it was considered as a remarkable risk factor for worldwide morbidity and the growing of mortality rates and causes with highly estimated domination of 10.7 million deaths [2]. Hence, the use of anti-hypertensive agents has a vital role in control of the resulting elevated blood pressure and its consequent illnesses [3]. According to recent researches [4], angiotensin receptor blockers (ARBs) proved more clinical effectiveness over angiotensin converting enzyme (ACE) inhibitors at lower adverse events. Two of the most widely used ARBs around the world are losartan (LOS) and valsartan (VLS). LOS and VLS (chemical structure figure 1) work on vasodilation of the arteries with small resistance, leading to attenuation of the whole peripheral resistance. Moreover, cardiac output and heart rate remain in normal manner, which means no possibility of postural hypotension. ARBs function is undoubtedly related to the improvement of baroreceptor job [5].

In 2018, the United States Food and Drug Administration (US-FDA) together with European Medicine Agency declared that the presence of carcinogenic nitrosamine derivatives as impurities in some ARBs known as ‘sartans’. More than 1400 products were recalled due to the presence of such carcinogenic impurities at levels exceeding the maximum intake limit (26.5 ng d^−1^) [6]. ARBs, containing tetrazole moieties in their structure including VLS and LOS, were suspected and traced for the presence of nitrosodiethylamine (NDEA) impurities which may originate during synthesis [7,8] (chemical structures figure 1). In 2020, the US-FDA released guidance for controlling the nitrosamine impurities in different pharmaceutical preparations. The development of methods for detection and quantification of NDEA impurities in pharmaceutical preparations containing ‘sartans’ has gained a momentum to replace the drawbacks of elder impurity profiling methodologies [9]. The use of ARBs was under concern as the US-FDA estimated that 1 case per 8000 patients receiving ARBs could develop cancer [10].

Meanwhile, the routine quality control (QC) analyses of pharmaceuticals using liquid chromatography (LC) techniques generate large amounts of organic waste; therefore, the use of green analytical chemistry (GAC) concepts is of great interest to minimize the amount of hazardous organic solvents generated daily worldwide or replace them with safer ones without loss in chromatographic performance [11]. Recently, green chromatographic approaches depending on the use of greener solvents are governed by two factors: cumulative energy demand and environmental health and safety assessments [12]. Cumulative energy demand refers to the emissions produced by each solvent into the environment as compared with its full life cycle; that is why it may be called life cycle assessment. Another GAC principle is to minimize energy utilization. That is why high energy instruments, such as UHPLC and MS/MS detectors are less favoured. Currently, testing options that were developed for nitrosamines' determination were almost completely based on chromatography coupled with MS/MS detection. The worldwide regulatory agencies exert robust efforts for this cause especially because more than 80% of drug manufacturing and QC is conducted in developing countries where MS/MS instrumentation is not conventionally available [10].

Although LOS and VLS were studied, in a dense way, alone or in combinations with other drugs using different analytical techniques [2,12–17], few literatures were reported for their determination with nitrosamine impurities. Determination of VLS and LOS with NDEA either alone or together was reported mainly using GC or LC with MS/MS detectors [18–27]. Despite the advantages of selectivity and sensitivity of mass detectors, they have two main defects. According to GAC, mass detectors are the lowest eco-friendly tools since they require high energies for their operation besides their incompatibility with wide range of mobile phase additives [28,29]. On the other hand, instrumentations related to them are expensive, which may not be suitable for routine QC analysis in low-budget laboratories and low-economy developing countries.

To our knowledge, only one article was reported recently for the determination of NDEA in VLS using conventional HPLC-UV [30]. However, the reported method did not consider the ecological aspects as it used a combination of ecologically hazardous reagents (formic acid, acetonitrile (ACN) and methanol (MeOH)). The reported method also covered only VLS, not covering LOS as presented in this research study.

The aim of the proposed study is to involve GAC aspects in developing a simple, green, sensitive and economic HPLC method for analysis of NDEA impurity in LOS and VLS to be feasible for routine QC analysis of the drugs and their pharmaceutical products.

## 2. Experimental

### 2.1. Equipment

Method development and analyses were performed on Waters Alliance 2695 HPLC system comprising a quaternary gradient pump, an autosampler, a column oven and a photodiode array detector 2996 (Waters, USA).

A RP-C18 symmetry column (75 × 4.6 mm, 3.5 µm) (Waters, Ireland) was used for the study.

### 2.2. Materials and reagents

VLS and LOS analytical standards were supplied and certified by EIPICo Pharmaceuticals, Egypt. NDEA was an analytical grade standard and was purchased from Sigma-Aldrich, Germany. ACN, ethanol (EtOH) and MeOH were supplied by Merck, Germany. Ammonium acetate (AMA) was purchased from Sigma Aldrich, (Germany). Pharmaceutical applications were done using tablet dosage forms purchased from the local Egyptian market. For VLS, Valsatens^®^ tablets containing 80 mg VLS per tablet produced by Amoun Pharmaceuticals Co. (batch no. 194749). LOS was determined in Cozaar^®^ tablets containing 50 mg LOS per tablet produced by Merck (batch no. T021907).

Deionized water was freshly prepared using Millipore water purification system.

### 2.3. Preparation of stock and standard solutions

First, working standard solutions of LOS, VLS and NDEA were prepared separately in EtOH at concentrations 10.0, 10.0 and 2.4 mg ml^−1^, respectively. To construct linearity curve, calibration standards were obtained by diluting working standards in a solvent mixture composed of EtOH and 0.02 M AMA (50 : 50, v/v). The calibration standards of NDEA were prepared at concentrations 0.5, 1.0, 2.0, 4.0, 6.0 and 10.0 µg ml^−1^. The calibration standards of VLS were prepared at 16.0, 24.0, 48.0, 80.0, 100.0 and 112.0 µg ml^−1^. LOS calibration standards were prepared at concentrations 0.5, 5.0, 10.0, 30.0, 50.0 and 75.0 µg ml^−1^.

Three QC standards, at low (QCL), intermediate (QCM) and high (QCH) concentration levels covering the specified ranges, were prepared from the working standard solution for testing accuracy, precision and robustness. QC standards were prepared by spiking the analytes under study in a placebo solution containing excipients that are commonly found in tablet formulations. Placebo solution was prepared in the solvent mixture and included starch, spray-dried lactose, carboxymethyl cellulose sodium, talc powder and methocel. NDEA QC standards were prepared at concentrations 1000, 2000 and 6000 ng ml^−1^, while those for VLS were prepared at concentrations 16, 24 and 80 µg ml^−1^. LOS QC standards were spiked at concentrations 10, 30 and 50 µg ml^−1^.

### 2.4. Chromatographic conditions

Chromatographic separation was performed using a conventional RP-C18 column. Gradient elution technique was performed using two mobile phase compositions (A and B). Mobile phase A was composed of 0.02 M AMA adjusted to pH 7.2 using dilute ammonia and acetic acid. Mobile phase B was pure EtOH. Gradient elution programme is listed in table 1. Mobile phase flow rate was 0.8 ml min^−1^ and the analytes were detected by UV detection at wavelength 230 nm. RP-C18 column was kept at 40°C all over the experiments. The injection volume was set at 20 µl.

**Table 1.** Gradiant programme for the proposed method for determination of LOS, VLS and NDEA.

time (min)	buffer (%)	EtOH (%)
0.0–3.0	90.0	10.0
3.0–8.0	90.0–40.0	10.0–60.0
8.0–9.30	40.0	60.0
9.30–10.30	40.0–90.0	60.0–10.0

### 2.5. Method validation

Method validation was performed according to ICH guidelines [31]. Six calibration standards of each drug and NDEA impurity were used to set the linearity and establish linearity equation relative to UV detector absorbance. The concentrations of each set of calibration standards are mentioned in §2.3. Three QC standards within the linearity ranges of the analytes under study were used to check the other validation parameters. From linearity data, limits of detection (LOD) and quantification (LOQ) for each analyte under study were calculated.

Accuracies were established by injecting the three QC standards in triplicates and calculated as a function of percentage of recoveries. Repeatability and intermediate precision were assessed using the QC standards for each analyte using recovery percentages when such standards were injected at different times within the same day and on three different days.

Method's robustness was assured by deliberately changing some factors of the chromatographic conditions. Temperature was changed by ±2°C. Percentage of organic phase, EtOH, was altered within the gradient programme by ±1%. The pH of the aqueous buffer was also altered by increments of ±0.1. Then these effects were studied on the recovery percentages of the QC standards of each analyte.

### 2.6. Method application

For determination of VLS and LOS in tablets of Valsatens^®^ and Cozaar^®^, 10 tablets of each drug dosage form were weighed and then powdered. The average weight equivalent to one tablet was transferred into 100 ml volumetric flask and volume completed with solvent mixture and sonicated for 10 min. The supernatant was filtered through a 0.45 µm filtration syringe. Then solution was diluted with the solvent mixture by transferring 5 ml into another 50 ml volumetric flask to be injected into HPLC for determination of its drug content.

## 3. Results and discussion

### 3.1. Method development

To establish a practical LC method for the simultaneous detection of NDEA in LOS and VLS products, the factors affecting chromatographic separation were to be chosen carefully. Two parameters were the main concerns: the efficiency of separations and considering GAC principles. Since column is the heart of HPLC where all separations are to be made, reversed phase C18, normal phase silica and bound cyano stationary phases were compared for efficiency in separation. RP-C18 was found the best option since it provided lower organic solvent consumption in better separation efficiency when compared with cyano-bound and normal stationary phase chromatography [2]. The chosen column's particle size was countered by column backpressure. As particle size decreases, the efficiency of separation increases; however, also the column backpressure magnifies so lower flow rates had to be effected [32]. The best-balanced separation efficiency as a function of column backpressure was obtained on RP-C18 column (75 × 4.6 mm, 3.5 µm).

Mobile phase composition represents a main ecological concern when developing new LC methodologies, since each conventional HPLC instrument can generate up to 0.5 l of organic waste daily [33]. Organic modifier was chosen according to its ecological impact. ACN although having unique separation characteristics, its ecological impact is high, besides its higher cost [34]. EtOH on the other hand had comparable efficiency, yet at lower ecological impact [35]. So, EtOH was chosen as organic modifier. Aqueous mobile phase additives were also considered. Such additives can be used for setting mobile phase pH in order to improve robustness, affect analyte's retention time based on pKa or to improve peak shape. Other additives act as ion-pairing reagents rendering some analytes as non-ionic molecules that can be retained by RP chromatography. Other mobile phase additives act as chiral selectors or chaotropic additives [36]. Screening aqueous additives, AMA was the best choice for its separation improving characteristics. AMA is volatile salt that can deactivate the active silanol groups within RP columns and acts as ion-pairing agent between analytes and stationary phase. AMA also can improve peak shape and affect retention time of analytes through adjusting its concentration in the mobile phase [37]. Moreover, its ecological impact is remarkable. The US National Fire Protection Association designates zero code for AMA in health and fire hazards, which indicates its environmental safety [38].

### 3.2. Method validation

When linearity was established, the working concentration range of NDEA had to be taken in consideration relative to LOS and VLS drugs' daily dose, to avoid the intake of over dose limits of NDEA. For instance, considering an average 320 mg daily intake of VLS tablets, the maximum limit for NDEA has to be 0.3 ppm [25]. Therefore, linearity range for NDEA was chosen to be at the lowest detectable limits (500–10 000 ng ml^−1^) relative to LOS and VLS linearity ranges described in table 2. The calibration curves were constructed between average detector responses relative to analyte's concentration. Table 2 shows calibration curve results across specified ranges. Correlation coefficients (*r*^2^) for both analytes indicated linear responses relative to concentration.

**Table 2.** Linearity results for the determination of LOS, VLS and NDEA using the proposed method.

parameter	LOS	VLS	NDEA
retention time (min ± s.d.)	3.3 ± 0.5	8.5 ± 0.4	9.4 ± 0.7
symmetry factor	0.87	0.83	0.89
linearity range (µg ml^−1^)	5.0–75.0	16.0–112.0	0.5–10.0
linearity equation	*y* = 168.5*x* + 241.8	*y* = 140.5*x* + 155.8	*y* = 214.3*x* + 7.7
correlation coefficient (*r*^2^)	0.9999	0.9999	0.9997
LOD (µg ml^−1^)	0.8	1.4	0.2
LOQ (µg ml^−1^)	2.4	4.5	0.5

The LODs and LOQs were calculated using the standard deviations (*σ*) and the slope (S) of the calibration curves. LODs were those equivalents to (3.3*σ*/S), while LOQs were those calculated from (10*σ*/S). Table 2 shows obtained results which prove sensitivity of the method for detection and quantification of NDEA in the presence of LOS and VLS.

**Figure 2.**
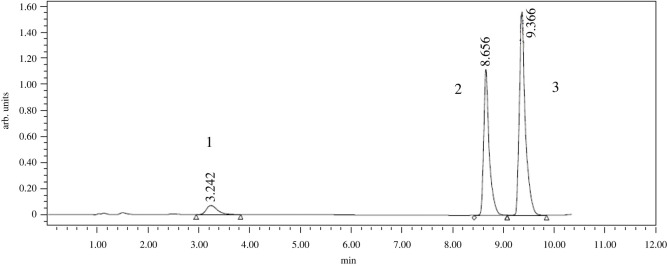
A typical chromatogram of 20 µl injection of standard solutions of (1) NDEA, (2) VLS and (3) LOS (spiked concentration 2.4, 20.0 and 10.0 µg ml^−1^, respectively) under proposed chromatographic conditions.

Specificity of the method defines the ability to determine chosen analytes in the presence of other excipients. As shown in figure 2, chromatograms of laboratory prepared mixture of LOS, VLS and NDEA in placebo mixture of excipients showed no interferences from any excipient.

Accuracy results express the closeness of the calculated recoveries to the actual spiked concentrations. Accuracy results for each drug in table 3 proved the large agreement between true and expected values.

**Table 3.** Accuracy and precision results of LOS, VLS and NDEA under the proposed method.

drug	spiked concentration (µg ml^−1^)	accuracy (recovery % ± s.d.)	intra-day precision (recovery % ± s.d.)	inter-day precision (recovery % ± s.d.)
NDEA	0.5	98.8 **±** 0.4	100.4 **±** 0.3	100.7 **±** 0.6
	1.0	100.9 **±** 0.5	100.8 **±** 0.7	101.3 **±** 0.8
	6.0	102.2 **±** 0.2	101.9 **±** 0.4	101.7 **±** 1.0
VLS	16.0	98.4 **±** 0.1	98.6 **±** 0.1	98.5 **±** 0.1
	24.0	101.9 **±** 0.1	101.2 **±** 0.3	101.9 **±** 0.8
	80.0	100.0 **±** 0.2	100.1 **±** 0.2	100.1 **±** 0.2
LOS	10.0	97.8 **±** 2.3	97.7 ± 0.7	95.0 ± 2.4
	30.0	102.6 **±** 0.8	102.4 ± 0.6	101.4 ± 0.4
	50.0	98.1 **±** 0.8	99.2 ± 0.2	98.9 ± 1.1

When precision was assessed within the same day (intra-day) and between different days (inter-day), results of repeatability and intermediate precisions (table 3) proved good agreement relative to actual concentrations.

Robustness of the method was assessed to demonstrate the constancy of the response against any deliberated minor changes in the experimental parameters. Table 4 shows that slight changes in the pH of the aqueous mobile phase part, slight changes in percentage of the organic part, or column temperature did not much affect the recovery results of QC standards.

**Table 4.** Robustness results of LOS, VLS and NDEA under the proposed method.

drug	spiked concentration (µg ml^−1^)	aqueous phase pH (±0.1)	temperature (±2°C)	EtOH% (±1%)
NDEA	6	99.2 **±** 0.7	99.9 ± 0.2	99.9 ± 0.1
VLS	24	100.0 ± 0.2	99.6 ± 0.5	100.2 ± 0.2
LOS	30	98.1 ± 0.8	100.0 ± 0.4	99.8 ± 0.6

### 3.3. Application of the proposed method

The method was used to determine VLS and LOS in their marketed dosage forms. The obtained results (table 5) were then compared with those obtained by a reference method [15]. The reference method estimated LOS and VLS by HPLC using RP-C18 column and isocratic mobile phase composed of 0.05 M potassium dihydrogen phosphate (pH 4.5) and ACN (55 : 45, v/v). The flow rate and the detection wavelength of the reference method were set at 1 ml min^−1^ and 210 nm, respectively. The student *t*-test and *F*-test results showed that there was no significant difference between results, proving method validity.

**Table 5.** Comparison between assay results of LOS and VLS in tablet dosage forms using the proposed methods to reported method.

	proposed method	reported method [17]	*t*-test	*F*-test
LOS	99.6 ± 0.5	100.1 ± 1.3	0.343	0.071
VLS	93.3 ± 0.3	93.7 ± 0.9	0.197	0.015

Average of six determinations per concentration (*n* = 6).

Calculated *t-* and *F*-test values are 2.776 and 19, respectively.

### 3.4. Comparison and evaluation against other reported methods

The assessment of newly developed analytical methodologies has become an important step during method development. Several assessment tools were introduced in the past few years including the analytical eco-scale [28], green analytical procedure index (GAPI) [39] and AGREE assessment tool [40].

The analytical eco-scale [28] metric assesses the analytical methodologies by assigning penalty points that depart from ideal (100 points) for steps within the method that does not meet the ideas of GAC. Penalty points are assigned mainly for two categories within the analysis: hazards and energy. Hazardous reagents are assessed according to type using the globally harmonized system (GHS) for classification and labelling of chemicals [41], and also assessed according to the amount used within the analysis. GHS uses nine pictograms and two signal words to characterize chemicals. Pictograms describe the type of hazard (for instance, corrosion, flame, skull, crossbones, etc.), while signals indicate the severity (i.e. ‘Danger’ for severe hazard and ‘Warning’ for less severe). Reagent label penalty points are calculated from multiplying the number of pictograms that it has on GHS by the signal's penalty points (2 for ‘Danger’ and 1 for ‘Warning’). Reagents' penalty points are finally then assessed from multiplying reagent classification penalty points by the consumed amount penalty points (1 for less than 10, 2 for 10–100, and 3 for more than 100 ml or gm reagent consumed). On the other hand, instrumentations are assessed for the total energy consumed throughout the analytical procedure, occupational hazard and waste generated. Energy is assessed for all instruments used during sample preparation as well as sample analysis. Penalty points for instruments operated at less than 0.1 kWh is 0, for 0.1–1.5 kWh is 1, and for more than 1.5 kWh is 2. The occupational hazard takes zero penalty points if there is analytical process hermetization and takes 3 penalty points if it causes emission of gases/vapour. The waste generated is calculated by multiplying penalty points departed from amount and treatment type. The waste volume/mass takes penalty points of 1, 3 or 5 if the amount generated is less than 1.0, 1.0–10.0 or more than 10.0 ml gm^−1^, respectively. On the other hand, treatment takes penalty points of 0, 1, 2 or 3, if the waste generated is recycled, naturally degrades, treated for passivation or not treated at all, respectively. Table 6 shows the analytical eco-score calculated for the proposed analytical methodology. The score of 90 indicates very good environmental safety of the proposed method.

**Table 6.** Assessment of the proposed method using the analytical eco-scale.

analytical eco-scale	penalty points
reagents	
ethanol (<10 ml)	6 × 1 = 6
AMA (<10 gm)	0
instrument	
energy	1
occupational hazard	0
waste (1–10 ml gm^−1^)	3 × 1 = 3
total penalty points	Σ10
analytical eco-scale score	100 – 10 = 90

Among those metrics, GAPI has demonstrated an easy, fast and reliable tool for investigating the greenness of analytical methods. GAPI considers all steps involved in the analysis, as indicated by its 15 pentagrams, from sampling, reagents and solvent used, sample preparation, instrumentation, as well as generated waste. AGREE introduces a clock-like graph which is divided into 12 significance principles, with the overall score represented with colour in the middle of the pictogram with values close to 1. The overall performance of each principle is introduced with the red-yellow-green colour scale where green represents the lowest ecological impact while the red colour indicates the higher impact. The dark green colour indicating that the assessed procedure is greener.

The proposed method was evaluated on GAPI and AGREE metrics against recently reported UHPLC-MS [25] and HPLC-UV [30] methods. As shown in table 7, the proposed method shows not only comparable, but even better ecological impact than the reported method. Although UHPLC has lower solvent consumption, especially that the chosen reported method used the green supercritical fluid as mobile phase; however, three main superiorities are demonstrated. First, MeOH used in the reported method has higher health hazard than EtOH when compared on NFPA code. Moreover, the drawbacks of UHPLC coupled with MS detection require higher energy for operation and additional sample treatment, filtration, step to avoid column blocking by any residues within the sample.

**Table 7.** Evaluation of the greenness on GAPI and AGREE assessment tools for the proposed method, and reference methods [26,30].

	proposed green HPLC method	reported method [26]	reported method [30]
technique	green HPLC-UV	UHPLC-MS	RP-HPLC- UV
mobile phase	0.02 M AMA adjusted to pH 7.2 and ethanol in gradient manner	CO_2_ as eluent A and methanol with 0.1% TFA as eluent B, added in gradient manner	solvent A: ACN, solvent B: water (pH 3.2 adjusted with formic acid) and solvent C: methanol in gradient manner
run time (min)	10	17	12
column	RP-C_18_ symmetry column (75 × 4.6 mm, 3.5 µm)	two HSS C18 SB columns (each 100 × 3.0 mm, 1.8 µm)	C_18_ (250 × 4.6 mm, 5 μm) column
GAPI assessment	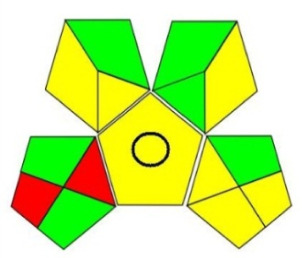	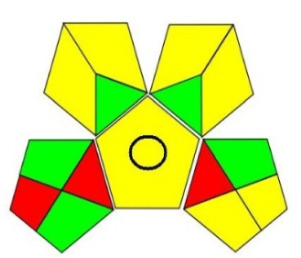	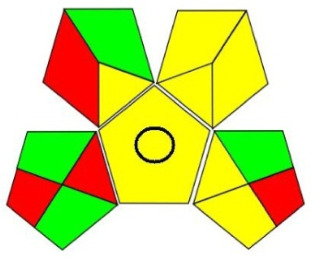
AGREE assessment	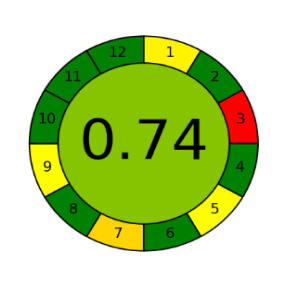	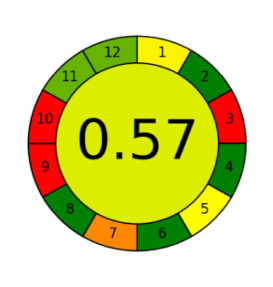	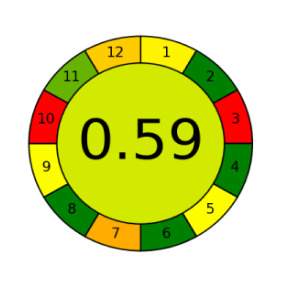

On one final view, the proposed method is cost-effective and requires less expensive instrumentation, lower energy consumption and sample preparation. The proposed method is even ecologically greener than reported methods and sensitive enough to be used for evaluation of marketed LOS and VLS products.

## 4. Conclusion

A new simple, fast and economic method was developed and validated for the determination of LOS and VLS in the presence of its NDEA carcinogenic impurity. Moreover, the proposed method is green and cost-effective, so it can be applied for routine day work analyses of drug together with its impurity with high predictability. The method was found to have the lowest ecological impact as assessed on both GAPI and AGREE greenness metrics.

## Data Availability

All supporting data are provided as electronic supplementary material (SM 1, 2, 3 and 4) [42].
